# Orchestration of Intracellular Circuits by G Protein-Coupled Receptor 39 for Hepatitis B Virus Proliferation

**DOI:** 10.3390/ijms21165661

**Published:** 2020-08-07

**Authors:** Kaku Goto, Hironori Nishitsuji, Masaya Sugiyama, Nao Nishida, Masashi Mizokami, Kunitada Shimotohno

**Affiliations:** Genome Medical Science Project, Research Center for Hepatitis and Immunology, National Center for Global Health and Medicine, Ichikawa, Chiba 272-8516, Japan; hironori.nishitsuji@fujita-hu.ac.jp (H.N.); msugiyama@hosp.ncgm.go.jp (M.S.); naonishida75@gmail.com (N.N.); mmizokami@hospk.ncgm.go.jp (M.M.); lbshimotohno@hospk.ncgm.go.jp (K.S.)

**Keywords:** GPR39, HBV, HCC, HSP, CEBPB, GLI

## Abstract

Hepatitis B virus (HBV), a highly persistent pathogen causing hepatocellular carcinoma (HCC), takes full advantage of host machinery, presenting therapeutic targets. Here we aimed to identify novel druggable host cellular factors using the reporter HBV we have recently generated. In an RNAi screen of G protein-coupled receptors (GPCRs), GPCR39 (GPR39) appeared as the top hit to facilitate HBV proliferation. Lentiviral overexpression of active GPR39 proteins and an agonist enhanced HBV replication and transcriptional activities of viral promoters, inducing the expression of CCAAT/enhancer binding protein (CEBP)-β (CEBPB). Meanwhile, GPR39 was uncovered to activate the heat shock response, upregulating the expression of proviral heat shock proteins (HSPs). In addition, glioma-associated oncogene homologue signaling, a recently reported target of GPR39, was suggested to inhibit HBV replication and eventually suppress expression of CEBPB and HSPs. Thus, GPR39 provirally governed intracellular circuits simultaneously affecting the carcinopathogenetic gene functions. GPR39 and the regulated signaling networks would serve as antiviral targets, and strategies with selective inhibitors of GPR39 functions can develop host-targeted antiviral therapies preventing HCC.

## 1. Introduction

Hepatitis B virus (HBV) remains a major pathogen responsible for chronic liver disease and hepatocellular carcinoma (HCC) claiming 887,000 lives annually [1,2,3]. Although prophylactic vaccines and direct-acting antivirals have been developed, HBV infection remains essentially incurable because of the complex and persistent strategy of viral replication [4,5,6]. The therapeutic potential of host-targeting agents (HTAs), on the other hand, is increasingly recognized due to viral dependence on and interference with host cellular factors for the viral life cycle and also carcinopathogenesis [7,8,9,10,11].

G protein-coupled receptors (GPCRs) constitute the largest family of druggable targets accounting for 30% of drugs on the market [12,13,14,15]. They are involved in nearly all physiological processes, as well as cancer and viral replication [16], providing targets germane to HTAs against HBV and HCC [17,18]. In the exploration of GPCRs to support HBV proliferation utilizing our new reporter system [19], we here identified G protein-coupled receptor 39 (GPR39) as the top host cellular factor candidate. GPR39 has been reported to regulate gastrointestinal motility and secretion, food intake, insulin secretion, tissue repair, apoptosis and synaptic transmission [20], mediating intracellular signaling pathways [21]. Coincidentally, the dysregulation and involvement of GPR39 in the development and progression of cancer have been also observed [22,23,24,25,26]. Here we strove to find additional modes of HBV proliferation through the properties of GPR39, also investigating novel features of the receptor through HBV replication machinery. Eventually, virus–host interactions reveal GPR39-centered intracellular networks utilized by HBV.

## 2. Results

### 2.1. A Screen of GPCRs for HBV Replication 

**Figure 1:**
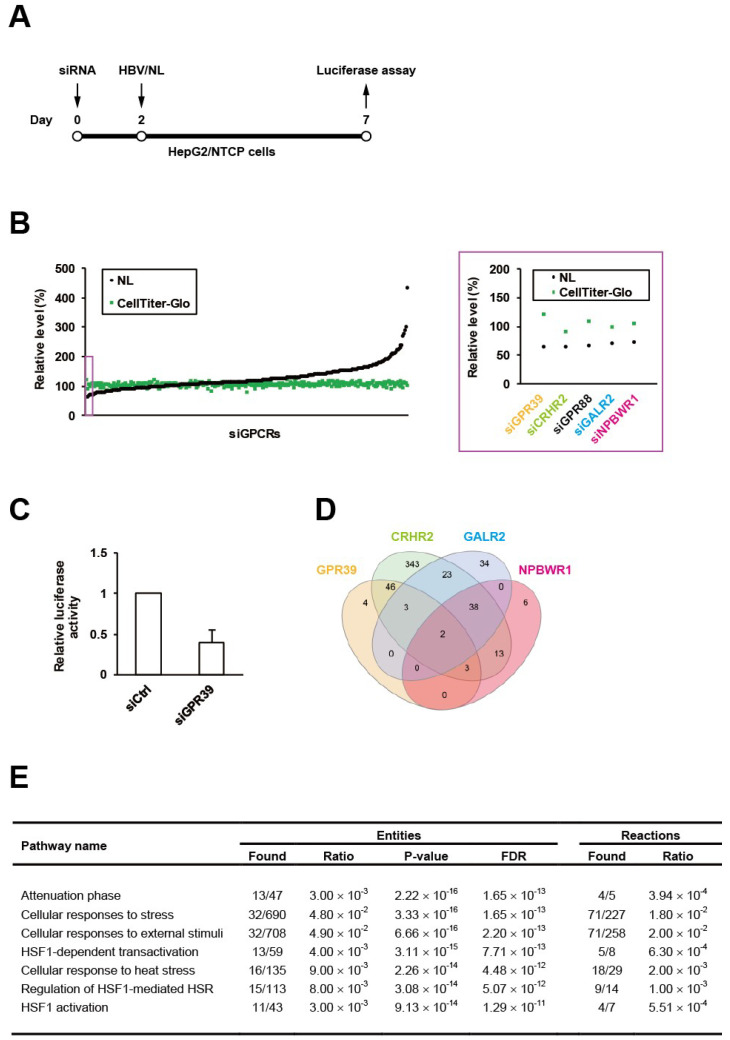
An RNAi screen of GPCRs for HBV proliferation. HepG2/NTCP cells were infected with HBV/NL at 5 days after the transfection of siRNAs to GPCRs for 2 days followed by the measurement of luciferase activities (**A**), and the relative levels of HBV and cell viability were indicated by NL and CellTiter-Glo, respectively, with the effects of knockdown of the top 5 genes highlighted by a purple rectangle and magnified on the right (**B**); the effect of GPR39 knockdown on HBV proliferation was demonstrated separately in a bar graph (**C**). (**D**) The Venn diagram of genes regulated by GPR39, CRHR2, GALR2 and NPBWR1. (**E**) Top significant pathways connected to the GPR39-GSK3B axis.

We have recently generated the reporter HBV carrying NanoLuc (NL), designated as HBV/NL [19], and here conducted an RNAi screen using a siRNA library for GPCRs in HepG2/NTCP cells according to our standardized protocol (Figure 1A). Among the 320 target genes, GPR39 was identified to be the top hit (Figure 1B), whose knockdown reduced the level of HBV/NL activity without affecting cell viability (Figure 1C). This was followed by the second and third hits corticotropin releasing hormone receptor 2 (CRHR2) and G protein-coupled receptor 88 (GPR88) (Figure 1B). To overview the global signaling dynamics, the downstream targets controlled by these receptors were analyzed by human GPCR network (hGPCRnet), a new web application for exploration of networks of GPCR signaling pathways [27]. The GPR39-connected genes through glycogen synthase kinase 3β (GSK3B) in hGPCRnet (Figure 1D) were shown to be primarily involved in heat shock response (HSR)-related pathways (Figure 1E) in Reactome, the database for cellular processes including signal transduction [28]. This trend was still observed (Supplementary Table S1) in the 46 genes commonly regulated by GPR39 and CRHR2 (Figure 1D) while GPR88 was not listed in hGPCRnet. The fourth and fifth hits galanin receptor 2 (GALR2) and neuropeptides B and W receptor 1 (NPBWR1) through melanocortin-4 receptor (MC4R) pertained to such independent pathways as related to microtubules (Supplementary Tables S2 and S3). Thus, the exemplary manifestation of GPCR-regulated cell circuits contributory to HBV replication represented by HSR-modulated [29] and microtubule-associated [30] networks as above underpinned the proviral impacts of GPCRs. 

Here, we focused on the most robust hit GPR39, a seven-transmembrane (TM) GPCR (Supplementary Figure S1A) governing critical physiological processes encompassing lipid metabolism, glucose homeostasis and cell proliferation via multiple intracellular signaling [20], and accordingly its molecular cellular impacts on HBV replication were investigated.

### 2.2. GPR39 Supports HBV Replication

**Figure 2:**
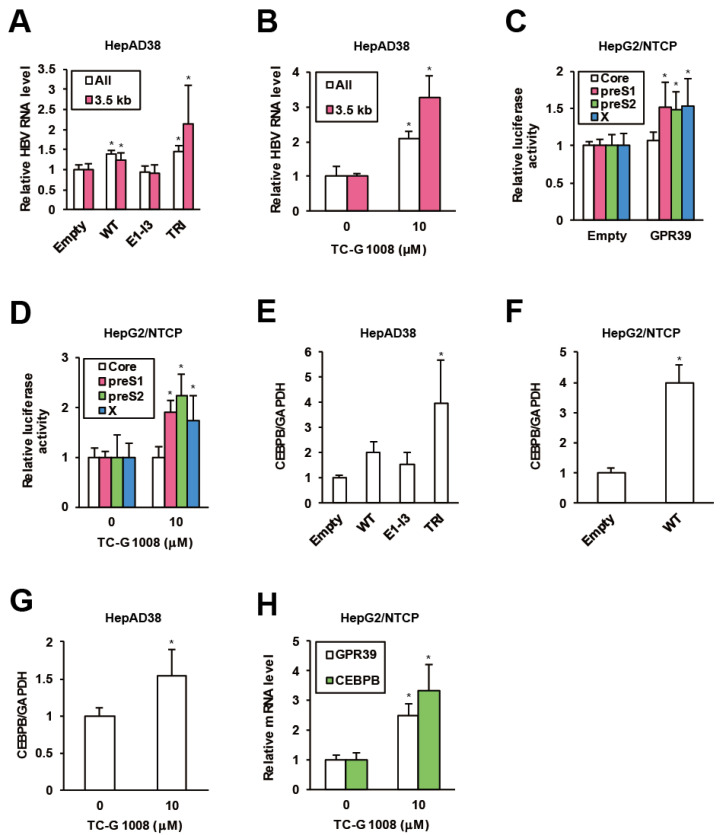
GPR39 supports HBV replication. (**A**) The effects of lentivirally overexpressed GPR39 proteins on HBV RNA. After preculture without tetracycline (Tet) for 7 days, HepAD38 cells were infected with lentiviruses for the empty control, WT, E1-I3 and TRI in the presence of Tet for 6 days. The relative levels of HBV RNAs were measured by qRT-PCR. (**B**) The effects of TC-G 1008 on HBV RNA levels in HepAD38 cells. After preculture without Tet for 7 days, the cells were treated with TC-G 1008 at 10 μM for 6 days in the presence of Tet. The relative RNA levels of were measured by qRT-PCR. (**C**) The effects of GPR39 on HBV promoter activities. The reporter plasmids carrying viral promoters were cotransfected with the expression plasmid of GPR39 into HepG2/NTCP cells for 2 days and relative luciferase activities were measured. (**D**) The effects of TC-G 1008 on HBV promoter activities. The reporter plasmids carrying viral promoters were transfected into HepG2/NTCP cells for 24 h and then treated with TC-G 1008 at 10 μM for 24 h, followed by the measurement of relative luciferase activities. (**E**) The effects of lentivirally overexpressed GPR39 proteins on CEBPB in HepAD38 cells. After preculture without Tet for 7 days, the cells were transduced with the lentiviruses for 6 days in the presence of Tet. The relative mRNA level of CEBPB was measured by qRT-PCR. (**F**) The effects of lentivirally overexpressed GPR39 on CEBPB in HepG2/NTCP cells. After the transduction with the lentiviruses for the empty control and GPR39 for 2 days, the relative mRNA level of CEBPB was measured by qRT-PCR. (**G**) The effects of TC-G 1008 on CEBPB expression in HepAD38 cells. The cells were treated as in (**B**) and the relative mRNA level was measured by qRT-PCR. (**H**) The effects of TC-G 1008 on CEBPB expression in HepG2/NTCP cells. After the treatment with TC-G 1008 at 10 μM for 2 days, the relative mRNA levels were measured by qRT-PCR. Statistical significances with *p* < 0.05 to controls were indicated by asterisks.

To assess the impacts of GPR39 on viral replication in detail, we harnessed the HBV-replicating HepAD38 cells and lentivirally overexpressed GPR39 proteins (Supplementary Figure S1A,B). The wild-type (WT) enhanced HBV RNA levels (Figure 2A) while a C-terminally truncated mutant E1-I3 (Supplementary Figure S1A) lacking receptor-activating and G-protein-interacting domains similarly to 5TM splice protein GPR39-1b [31], did not exert the effect (Figure 2A). In contrast, the mutant TRI with point mutations at three residues, C108A, C191A and D313A (Supplementary Figure S1A) reported to confer high constitutive activities [32,33] further boosted the viral RNA levels (Figure 2A). Thus through the correlation of the receptor activity and viral RNAs, GPR39 was suggested to facilitate HBV replication. Indeed, TC-G 1008, a reported agonist [34] and expression inducer [35] of GPR39, upregulated viral RNA levels in HepAD38 cells (Figure 2B) as well as HepG2/NTCP cells infected with wild type HBV (Supplementary Figure S2A), simultaneously enhancing GPR39 expression itself (Supplementary Figure S2B). 

To elucidate the mechanism of the enhancement of HBV RNA levels by GPR39, we assessed viral transcription. In HepG2/NTCP cells, GPR39 was transiently overexpressed in the presence of reporter plasmids encoding luciferase gene downstream of individual viral promoter sequences [36]. The promoter activities of PreS1, PreS2 and X were enhanced while that of pregenome/PreCore was not (Figure 2C), which was supported by knockdown of GPR39 (Supplementary Figure S2C). In parallel, the promoter activity showed a similar profile in the presence of TC-G 1008 (Figure 2D), and was confirmed to be boosted by GPR39. Hence, representative liver-enriched transcription factors reported to support HBV gene expression, such as hepatocyte nuclear factors (HNFs) and CCAAT/enhancer binding proteins (CEBPs) [37], were investigated. The overexpression of GPR39 and its active mutant significantly heightened the expression of CEBP-β (CEBPB) above all in HepAD38 cells (Figure 2E and Supplementary Figure S3A), which was confirmed in HepG2/NTCP cells (Figure 2F and Supplementary Figure S3B). TC-G 1008 likewise elevated the expression of CEBPB in both cell lines (Figure 2G,H).

### 2.3. GPR39 Activates HSR

**Figure 3:**
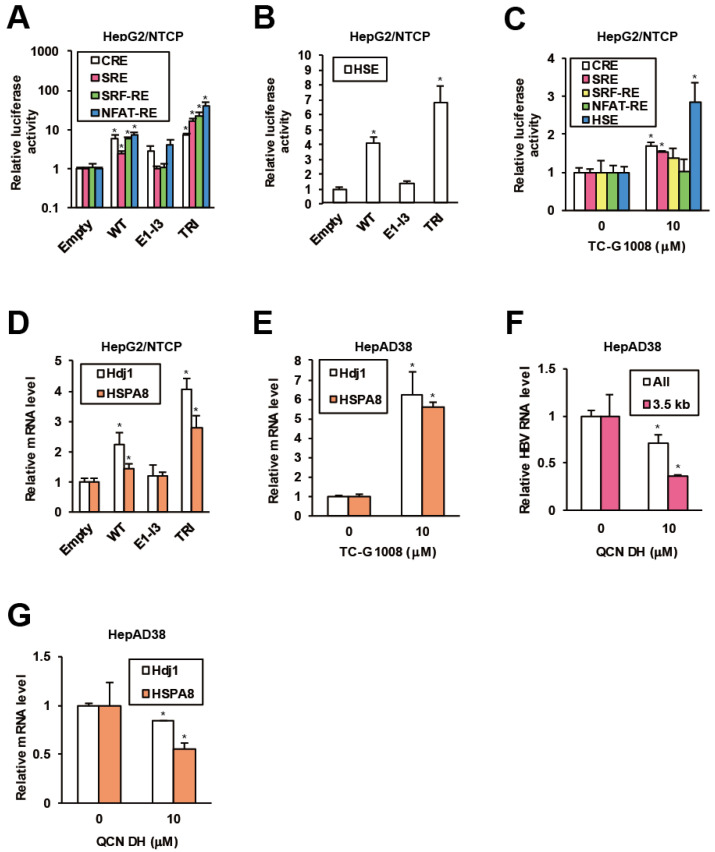
GPR39 facilitates HSR and HSP expression. (**A**) The effects of GPR39 on intracellular signaling. HepG2/NTCP cells were cotransfected with reporter plasmids for CRE, SRE, SRF-RE and NFAT-RE and expression plasmids for GPR39 proteins for 2 days, followed by the measurement of relative luciferase activities. (**B**) The effects of GPR39 on HSE. HepG2/NTCP cells were transfected with the HSE reporter and the GPR39 expression plasmids for 2 days, followed by the measurement of relative luciferase activities. (**C**) The effects of TC-G 1008 on HSE. Twenty four hours after the transfection of the reporter plasmids HepG2/NTCP cells were treated with TC-G 1008 at 10 μM for 24 h, followed by the measurement of relative luciferase activities. (**D**) The effects of lentivirally overexpressed GPR39 proteins on Hdj1 and HSPA8 in HepG2/NTCP cells. The cells were transduced with the lentiviruses for 2 days and the relative mRNA levels were measured by qRT-PCR. (**E**) The effects of TC-G 1008 on the expression of Hdj1 and HSPA8 in HepAD38 cells. After the treatment with TC-G 1008 at 10 μM for 6 days in the presence of Tet following preculture without Tet for 7 days, the relative mRNA levels were measured by qRT-PCR. (**F**) The effects of QCN on HBV RNA levels. After preculture without Tet for 7 days, HepAD38 cells were treated with QCN DH at 10 μM for 6 days in the presence of Tet. The relative RNA levels were measured by qRT-PCR. (**G**) The effects of QCN on the expression of Hdj1 and HSPA8. HepAD38 cells were treated as in (**F**) and the relative mRNA levels were measured by qRT-PCR. Statistical significances with *p* < 0.05 to controls were indicated by asterisks.

Regulatory effects of GPR39 on major GPCR signaling pathways were previously demonstrated in mouse cell lines [38,39], and accordingly we evaluated them in hepatocytes. In HepG2/NTCP cells, overexpression of the WT GPR39 accentuated G protein-mediated cascades monitored by activities of luciferase downstream of response elements such as cAMP response element (CRE), nuclear factor of activated T-cells response element (NFAT-RE), serum response element (SRE) and serum response factor response element (SRF-RE). The hyperactive mutant TRI further enhanced the effects, while the C-terminally truncated mutant E1-I3 did not (Figure 3A). Then we explored the effects of GPR39 on heat shock element (HSE) as indicated by the pathway analysis on GPR39-connected genes (Figure 1E and Supplementary Table S1), and HSE significantly responded to GPR39 overexpression (Figure 3B). This induction was also observed by the treatment with TC-G 1008 (Figure 3C). Meanwhile, the effects of the truncated mutants E2-I4, E3-I4 and E4-I4 (Supplementary Figure S1A) void of N-terminal domains were decreased in proportion to the molecular length (Supplementary Figure S3C).

Based on the indication of GPR39-stimulated HSR, involvement of HSPs in proviral network of GPR39 was strongly suggested. To test this hypothesis, the effect of lentiviral overexpression of GPR39 on the expression of HSPs as well as heat shock factors (HSFs) was examined. Of major HSPs reportedly supportive of HBV replication [40,41], DnaJ heat shock protein family (Hsp40) member B1 (Hdj1) and heat shock 70 kD protein 8 (HSPA8) demonstrated highly induced expression, particularly in the presence of TRI in HepG2/NTCP cells (Figure 3D) over the others (Supplementary Figure S3D). Also the expression of Hdj1 and HSPA8 was remarkably enhanced by TC-G 1008 in HepAD38 cells (Figure 3E and Supplementary Figure S3E), consistently indicating GPR39-modulated HSR. In addition, quercetin dihydrate (QCN DH), a reported inhibitor of HBV replication through transcriptional inhibition of HSPs [41], reduced viral RNA levels in HepAD38 cells (Figure 3F), where Hdj1 and HSPA8 were transcriptionally downregulated (Figure 3G) similarly to the other HSPs (Supplementary Figure S3F). Collectively, GPR39 was suggested to activate HSR leading to HSPs expression and viral replication. 

### 2.4. GPR39-Targeted Hedgehog (HH) Signaling against HBV Replication 

**Figure 4:**
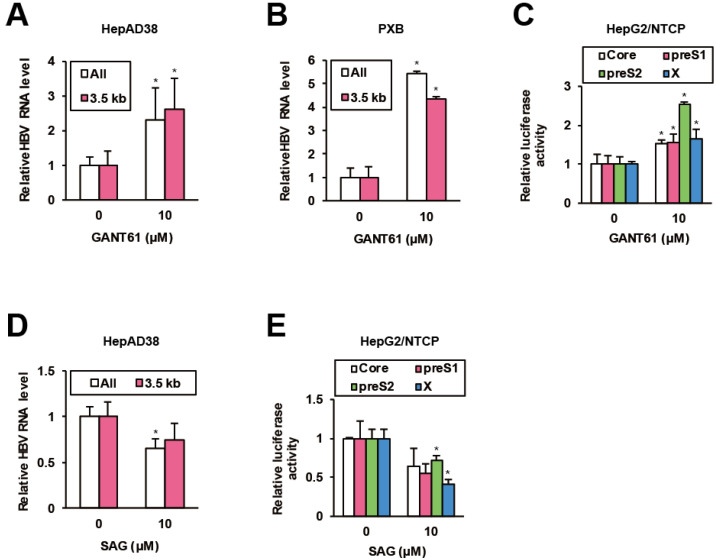
HH signaling suppresses HBV. (**A**) The effects of GANT61 on HBV RNA levels in HepAD38 cells. After preculture without Tet for 7 days, the cells were treated with GANT61 at 10 μM for 6 days in the presence of Tet, and the relative RNA levels were measured by qRT-PCR. (**B**) The effects of GANT61 on HBV RNA levels in PXB cells. Twenty four hours after the infection with HBV, PXB cells were treated with GANT61 at 10 μM for 4 days. The relative RNA levels were measured by qRT-PCR. (**C**) The effects of GANT61 on activities of viral promoters. Twenty four hours after the transfection of the reporter plasmids carrying viral promoters, HepG2/NTCP cells were treated with GANT61 at 10 μM for 24 h, and the relative luciferase activities were measured. (**D**) The effects of SAG on HBV RNA levels in HepAD38 cells. As in (**A**), the cells were treated with SAG at 10 μM and the relative RNA levels were measured by qRT-PCR. (**E**) The effects of SAG on activities of viral promoters. As in (**C**), the cells were treated with SAG at 10 μM, and the relative luciferase activities were measured. Statistical significances with *p* < 0.05 to controls were indicated by asterisks.

A recent chemical biological study on HH inhibitors discovered that GPR39 impinges on HH signaling through glioma-associated oncogene homologue (GLI) transcription factors [42]. Hence we investigated the effects of a GLI inhibitor GANT61 [43] on HBV replication. In HepAD38 cells, GANT61 significantly elevated viral RNA levels (Figure 4A), which was confirmed in HBV-infected PXB cells (Figure 4B), human hepatocytes isolated from chimeric mice with a humanized liver [44]. Simultaneously, GANT61 stimulated viral promoter activities in HepG2/NTCP cells (Figure 4C). Conversely, smoothened (SMO) agonist (SAG), an HH signaling agonist [45], suppressed viral RNA levels in HepAD38 cells (Figure 4D) and viral promoter activities in HepG2/NTCP cells (Figure 4E). The data indicated that GPR39-targeted GLI signaling limited HBV replication. 

### 2.5. GPR39-Orchestrated Crosstalks among CEBPB, HSPs and HH Signaling for HBV

**Figure 5:**
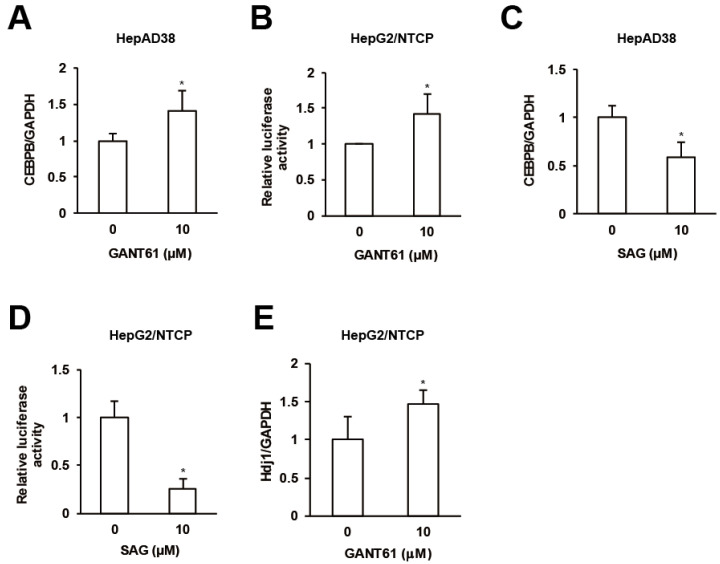
Crosstalks orchestrated by GPR39. (**A**) The effect of GANT61 on CEBPB expression. After the treatment of HepG2/NTCP cells with GANT61 at 10 μM for 2 days, the relative mRNA levels were measured by qRT-PCR. (**B**) The effect of GANT61 signaling on HSR. HepG2/NTCP cells were transfected with the reporter plasmid for HSE for 24 h, followed by the treatment with either GANT61 at 10 μM for 24 h, and the relative luciferase activities were measured. (**C**) The effect of SAG on CEBPB expression. As in (**A**), the cells were treated with SAG at 10 μM and the relative mRNA levels were measured by qRT-PCR. (**D**) The effect of SAG on HSR. As in (**B**), the cells transfected with the reporter plasmid for HSE were treated with SAG at 10 μM, and the relative luciferase activities were measured. (**E**) The effect of GANT61 on the expression of Hdj1. After the treatment of HepG2/NTCP cells with GANT61 at 10 μM for 2 days, the relative mRNA level of Hdj1 was measured by qRT-PCR. Statistical significances with *p* < 0.05 to controls were indicated by asterisks.

Finally we interrogated interrelations among GPR39 targets. GANT61 enhanced the expression of CEBPB in HepAD38 cells (Figure 5A) and HSR in HepG2/NTCP cells as assessed in Figure 3C (Figure 5B) in contrast to SAG (Figure 5C,D). The expression of HSPs including Hdj1 was also elevated by GANT61 in HepG2/NTCP cells (Figure 5E and Supplementary Figure S3G). The results suggested that HH signaling molecules suppressed CEBPB expression and HSR.

## 3. Discussion

Recent studies growingly revealed the significance of host cellular factors in the viral life cycle [46] and also carcinopathogenesis [47,48]. Exemplarily, cyclophilin (CyP) has been demonstrated to be a critical host cellular factor [49]. The CyP inhibitor CRV431 inhibited liver HBV DNA and hepatitis B surface antigen in vivo [50] and CyPA was overexpressed in HCC promoting cell cycle [51]. Our study also uncovered that GPR39 supported HBV replication concomitantly regulating host cellular signaling and networks. In fact, GPR39 was recently reported as a prognostic predictor of HCC negatively correlated with survival rates [52].

GPR39 intracellularly transmits signals to stimulate multiple response elements for downstream gene expression [53]. Based on the GPR39-enhanced HBV RNA level, CEBPB was identified to be modulated by GPR39, and at least CRE can mediate CEBPB expression as exhibited in macrophage [54,55]. HBV increases the expression of CEBPB [56] employing CRE [57], raising questions about possible engagement of GPR39. Additionally, the pathway analyses (Figure 1E) upheld by antiviral effects of HSP inhibitors (Supplementary Figure S3H) in our recent chemical screen [36] led to the discovery of GPR39-induced HSR and expression of HSPs, well-known host cellular factors for viral replication [58]. Possible GPR39-mediated regulation of GSK3B, a reported GPR39 interactor [59] would be interesting, since GSK3B is able to repress HSF1 [60]. So far a putative GPCR was required for HSR in worms [61], and molecular assessment of the GPR39-driven HSR in hepatocytes is warranted in accordance with the pathway analysis (Figure 1E and Supplementary Table S1). On the other hand, the upregulation of HBV genome RNA level by GPR39 was irrelevant to the promoter activity of pregenome/PreCore though possibly bolstered by that of HBx (Figure 2C,D) [62]. Alternatively, HSPs-facilitated reverse transcription and assembly [63,64] could lead to increase in HBV DNA through recycling and accumulation of pgRNA protected from nuclease by encapsidation. Moreover, other host cellular factors and mechanisms under the control of GPR39 could underlie the phenomenon, which needs exploring and examining in tandem with kinetic analyses of viral DNAs and proteins in detail beyond the current study. With respect to the molecular features of GPR39, the importance of the C-terminal tail in signaling was indicated by E1-I3 (Figure 3A) lacking TM6 and TM7 associated with 7TM receptor activation similarly to 5TM splice protein GPR39-1b [31], and the impacts of functional residues [32,33] were confirmed in hepatocytes. Indeed, C108A reportedly abrogated glycosylation [32] as was observed in the reduction in the posttranslational modifications of TRI (Supplementary Figure S1B), which demonstrated elevated activities reflected in signaling transduction (Figure 3A,B) as well as HBV replication (Figure 1A). In addition, we found the N-terminal domains played significant roles (Supplementary Figure S1A) in signaling [65], in agreement with the observation of the disulfide bridge between Cys11 and Cys191 maintaining the activity of GPR39 [32]. Inter- and intra-molecular interactions [66,67] are thus further indicated, warranting the exploration of additional interactors, modifications and structures of GPR39. The discovery of specific molecular sites and features for signaling would lead to biological understanding of the receptor and selective modulation of individual pathways.

CEBPB not only facilitates viral transcription [68,69] but also contributes to HCC cell proliferation and invasion [70] and predating pathogenic events subsuming inflammation, endoplasmic reticulum stress and hepatic steatosis when highly expressed [71]. Hence, the control of CEBPB would inhibit both the virus and carcinogenesis [72]. Likewise, HSPs not only expedite viral replication [40,41] but also participate in cancers including HCC [73,74]. Indeed the upregulation of HSPA8 expression was associated with vascular endothelial growth factor in HCC patients [75]. From this perspective, QCN could be fit for interventional purpose as the antiviral natural products exemplified by flavonoids and alkaloids possess anti-HCC properties [76,77]. QCN was even demonstrated to inhibit the transcriptional activity of CEBPB as well [78], and targeting GPR39-governed host cellular factors such as HSPs and CEBPB concurrently would suppress both HBV and HCC.

In turn, HH signaling, a recently reported inhibitory target of GPR39 [42], was indicated to suppress HBV replication. HH signaling has been demonstrated to play a substantial role in hepatocarcinogenesis and HCC progression [79,80,81,82]. On the other hand, AKT, an oncogenic kinase for hepatocyte survival, was activated by HBx and inhibited HBV replication, indicative of viral tactics for immune evasion and persistent production of progeny at the cost of high viral replication [83]. Conceivably, such a balancing mechanism as above via HH signaling can be inferred, and downstream molecules responsible for the inhibition of HBV, expected to be discovered in exploratory studies, would be exploited as antiviral targets.

**Figure 6:**
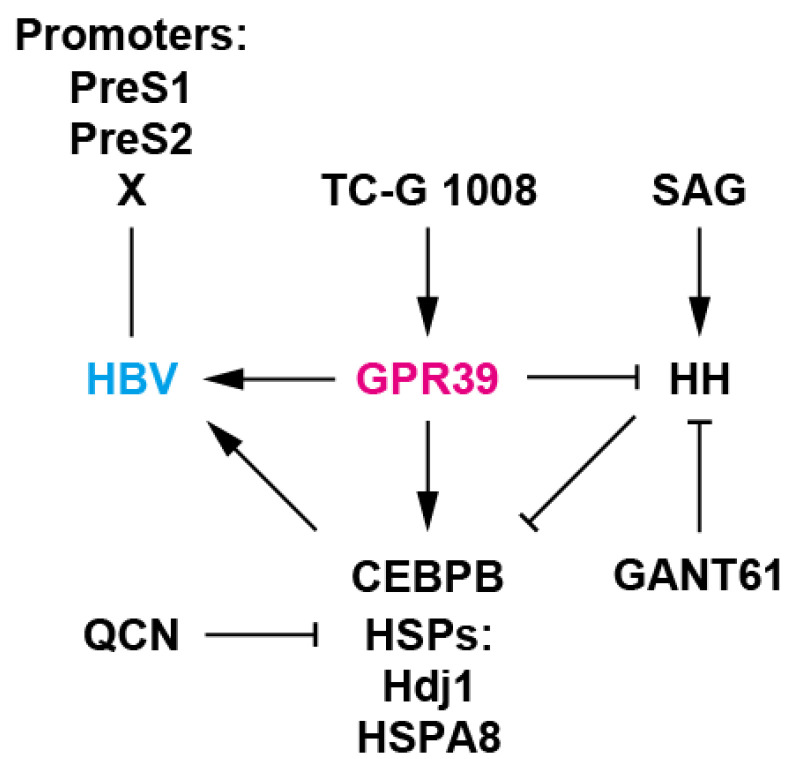
A working model of GPR39-centered network. GPR39 supported HBV replication, enhancing viral promoter activities as well as RNA levels and expression of HSPs such as Hdj1 and HSPA8 and CEBPB, well-known host cellular factors for HBV. TC-G 1008 is an agonist of GPR39 [34] and QCN is an inhibitor of HSP transcription and HBV [41]. Meanwhile, HH signaling, a GPR39 target, suppressed HBV replication as well as expression of HSPs and CEBPB. SAG is an HH signaling agonist [45] and GANT61 is a GLI inhibitor [43]. GPR39 thus orchestrates intracellular circuits, providing a proviral environment, and alteration in expression is conducive to pathogenic effects [52] as observed with HCC-related GPCRs [17].

Crosstalks among the signaling and factors are also noteworthy. The GANT61-enhanced expression of CEBPB and HSPs implied their suppression by GLI, thereby explaining its antiviral effects. CEBPB is also indicated to be a transcription factor for the expression of itself [84] and GPR39 [31], thereby presumably extrapolating CEBPB autostimulation-boosted reciprocal upregulation between CEBPB and GPR39. Notably, TC-G 1008, an agonist of GPR39 [34] enhanced the expression of GPR39 (Figure 2B,H) in agreement with the observation in vivo [35]. This can be caused by CEBPB upregulated by TC-G 1008-mediated GPR39 agonism (Figure 2G,H). After all, the network is orchestrated by GPR39, a GLI repressor [43], providing the environment supportive of HBV (Figure 6). Interestingly, GPR39 is transcriptionally regulated by HNF1A and HNF4A [31,85], which are required for maintenance of hepatocyte function and HBV lifecycle [37]. Therefore, the proper control of HNFs could effectively suppress viral gene expression through intracellular environmental and transcriptional mechanisms. 

Based on these findings, the modulation of GPR39 would present dually effective strategies against both HBV and HCC. However, collateral effects on HH signaling need to be averted for management of the HCC risk, and HH antagonism [86,87] could be considered simultaneously. More advanced and deepened knowledge of GPR39 encompassing its novel physiological roles and downstream effectors yet to be investigated and defined would lead to the development of functionally selective GPR39 inhibitors without off-target side effects. 

The identification of GPR39 as a host cellular factor for HBV clarified new properties and orchestrated circuits of the receptor. Further analyses on GPR39 would enable greater comprehension of HBV replication/pathogenesis and GPCR signaling/networks, serving to create novel HTAs and preventive and therapeutic options for HBV infection and HCC.

## 4. Materials and Methods

### 4.1. Compounds and Cells

TC-G 1008, GANT61, SAG dihydrochloride, and QCN DH were purchased from Tocris Bioscicence (Bristol, UK), Wako Pure Chemicals (Osaka, Japan), Sigma-Aldrich (St. Louis, MO, USA), and Nacalai Tesque (Kyoto, Japan), respectively. Antibodies to FLAG and β-actin (ACTB) were purchased from Sigma-Aldrich. HepG2/NTCP and HepAD38 cells were cultured as reported previously [36]. PXB cells were purchased from Phoenix Bio (Hiroshima, Japan). All human cell lines have been authenticated using STR profiling in Jun 2019, and all experiments were performed with mycoplasma-free cells.

### 4.2. Plasmids

Following the amplification by the primers Nest-GPR39-F and Nest-GPR39-R, using cDNAs from Huh7 cells, coding sequences of GPR39 were amplified by the primers, EcoRI-GPR39-F and XbaI-GPR39-R, and cloned into p3xFLAG-CMV-14 (Sigma-Aldrich), generating pCMV14-GPR39-3xFL. For pCMV14-GPR39(E1-I3)-3xFL, pCMV14-GPR39(E2-I4)-3xFL, pCMV14-GPR39(E3-I4)-3xFL, and pCMV14-GPR39(E4-I4)-3xFL, primer sets EcoRI-GPR39-F plus XbaI-GPR39-I3-R, EcoRI-GPR39-E2-F plus XbaI-GPR39-R, EcoRI-GPR39-E3-F plus XbaI-GPR39-R, and EcoRI-GPR39-E4-F plus XbaI-GPR39-R were used, respectively, and pCMV14-GPR39-3xFL as templates (Supplementary Table S4). On the other hand, the primers GPR39-C108A-F and GPR39-C108A-R were used for pCMV14-GPR39(C108A)-3xFL, which served as templates for the amplification using the primers GPR39-C191A-F and GPR39-C191A-R, generating pCMV14-GPR39(C108/191A)-3xFL. Subsequently, pCMV14-GPR39(TRI)-3xFL was generated using the primers GPR39-D313A-F and GPR39-D313A-R. For pCMV14-HBx-3xFL, the primers EcoRI-HBx-F1 and XbaI-HBx-R2 were used.

### 4.3. GPCR Pathway Analysis 

Genes connected to individual GPCRs were selected by hGPCRnet networking the receptors, transducers and effectors of GPCR signalings [27] and the Venn diagram was generated by InteractiVenn [88]. Pathways related to groups of genes were calculated by Reactome [28].

### 4.4. Luciferase Assay

Firefly luciferase activity was monitored by a dual-luciferase reporter assay system (Promega, Madison, WI, USA) as described previously [89]. Luciferase reporters carrying HBV promoters were generated previously [36] and pGL4.29, pGL4.30, pGL4.33, pGL4.34, pGL4.41 were purchased from Promega. The luciferase activities of the reporters were normalized to those of pRL-TK cotransfected.

### 4.5. Quantitative Reverse Transcription-Polymerase Chain Reaction

Relative mRNA levels and HBV RNA levels were quantified as described previously [89] using the primer sets (Supplementary Table S5), with the normalization to the level of *GAPDH*.

### 4.6. Western Blot

Total protein was resolved by SDS-PAGE and subjected to western blotting as described previously [89].

### 4.7. Lentivirus

Coding sequences of pCMV14-GPR39-3xFL, pCMV14-GPR39(E1-I3)-3xFL, and pCMV14-GPR39(TRI)-3xFL were amplified using primers GPR39-INF-F and GPR39-INF-R, and inserted into a multi-cloning site of pCSII-CMV-MCS. Lentiviral infection was performed as described previously [90].

### 4.8. RNAi

SiRNA-mediated knockdown of GPR39 in the HBV NL system was performed as described previously [19].

### 4.9. Statistical Analysis

The bar graphs are presented as means ± SD. *T*-tests were used and statistical significances with *p* < 0.05 to controls were indicated by asterisks.

## Supplementary Materials

Supplementary Materials can be found at https://www.mdpi.com/1422-0067/21/16/5661/s1. Figure S1: GPR39 expression, Figure S2: Effects of GPR39 modulation on HBV and HSR, Figure S3: Effects of overexpression and pharmacological modulation of GPR39 on gene expression, HSR and HBV, Table S1: The top five significant pathways connected to the GPR39-GSK3B axis and CRHR2, Table S2: The top 10 significant pathways connected to GALR2, Table S3: The top 10 significant pathways connected to the NPBWR1-MC4R axis, Table S4: Primers for construction of plasmids, Table S5: Primers for qRT-PCR.

## Abbreviations

HBV	hepatitis B virus
HCC	hepatocellular carcinoma
GPCR	G protein-coupled receptor
HSP	heat shock protein
CEBPB	CCAAT/enhancer binding protein-β
HTA	host-targeting agent
GPR39	G protein-coupled receptor 39
NL	NanoLuc
CRHR2	corticotropin releasing hormone receptor 2
GPR88	G protein-coupled receptor 88
hGPCRnet	human GPCR network
GSK3B	glycogen synthase kinase 3β
HSR	heat shock response
GALR2	galanin receptor 2
NPBWR1	neuropeptides B and W receptor 1
MC4R	melanocortin-4 receptor
CRE	cAMP response element
NFAT-RE	nuclear factor of activated T-cells response element
SRE	serum response element
SRF-RE	serum response factor response element
HSE	heat shock element
Hdj1	DnaJ heat shock protein family member B1
HSPA8	heat shock 70 kD protein 8
QCN DH	quercetin dihydrate
HH	hedgehog
GLI	glioma-associated oncogene homologue
SAG	smoothened agonist
CyP	cyclophilin
HSF	heat shock factor
