# Supplementary material for: Orchestration of Intracellular Circuits by G Protein-Coupled Receptor 39 for Hepatitis B Virus Proliferation
Source: Int J Mol Sci. 2020 Aug 7;21(16):5661. doi: 10.3390/ijms21165661 (PMC7460832; doi:10.3390/ijms21165661)
Supplement: Supplementary file 1 [file ijms-21-05661-s001.zip › Supplmentary Materials_3.pdf]

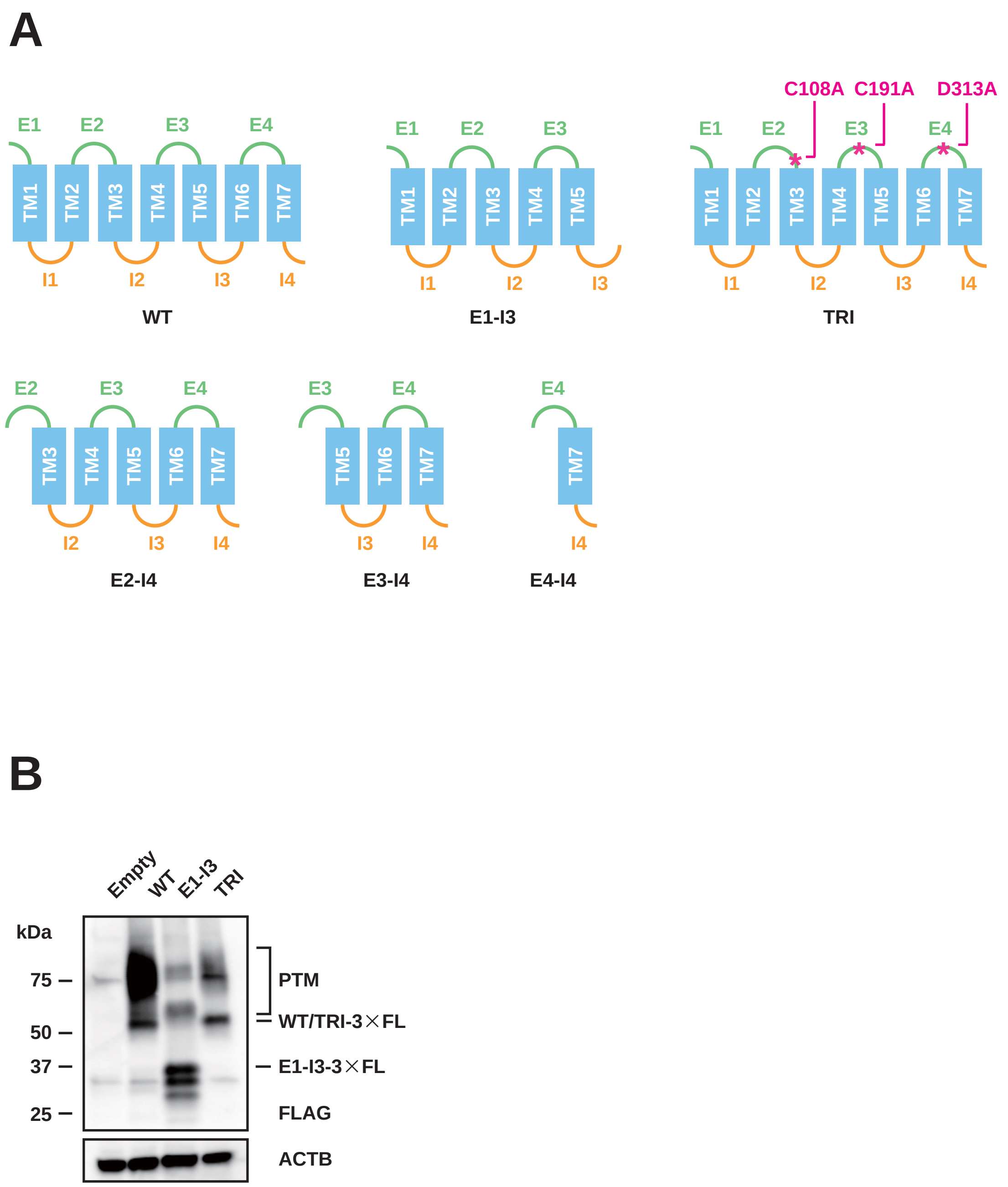

Supplementary Figure S1

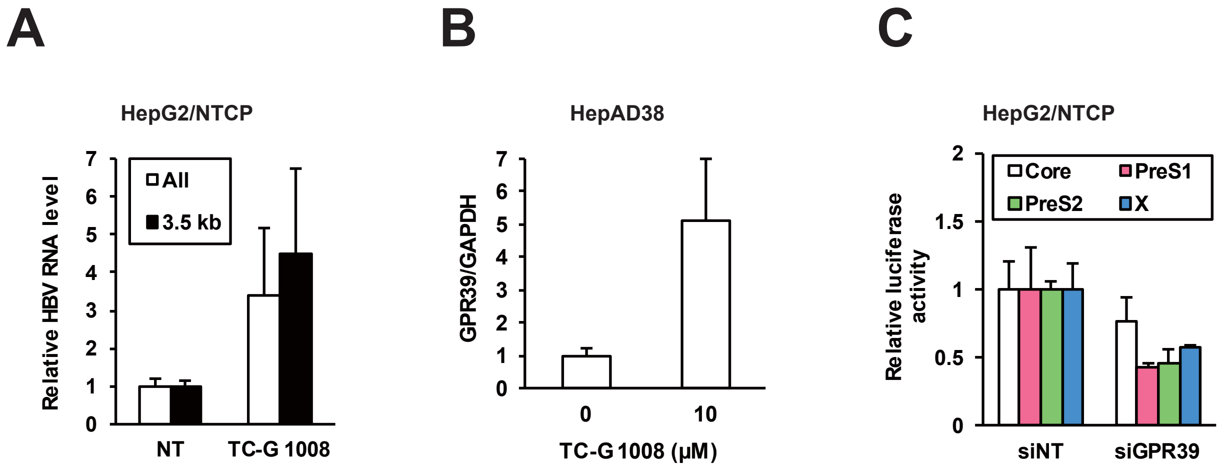

Supplementary Figure S2

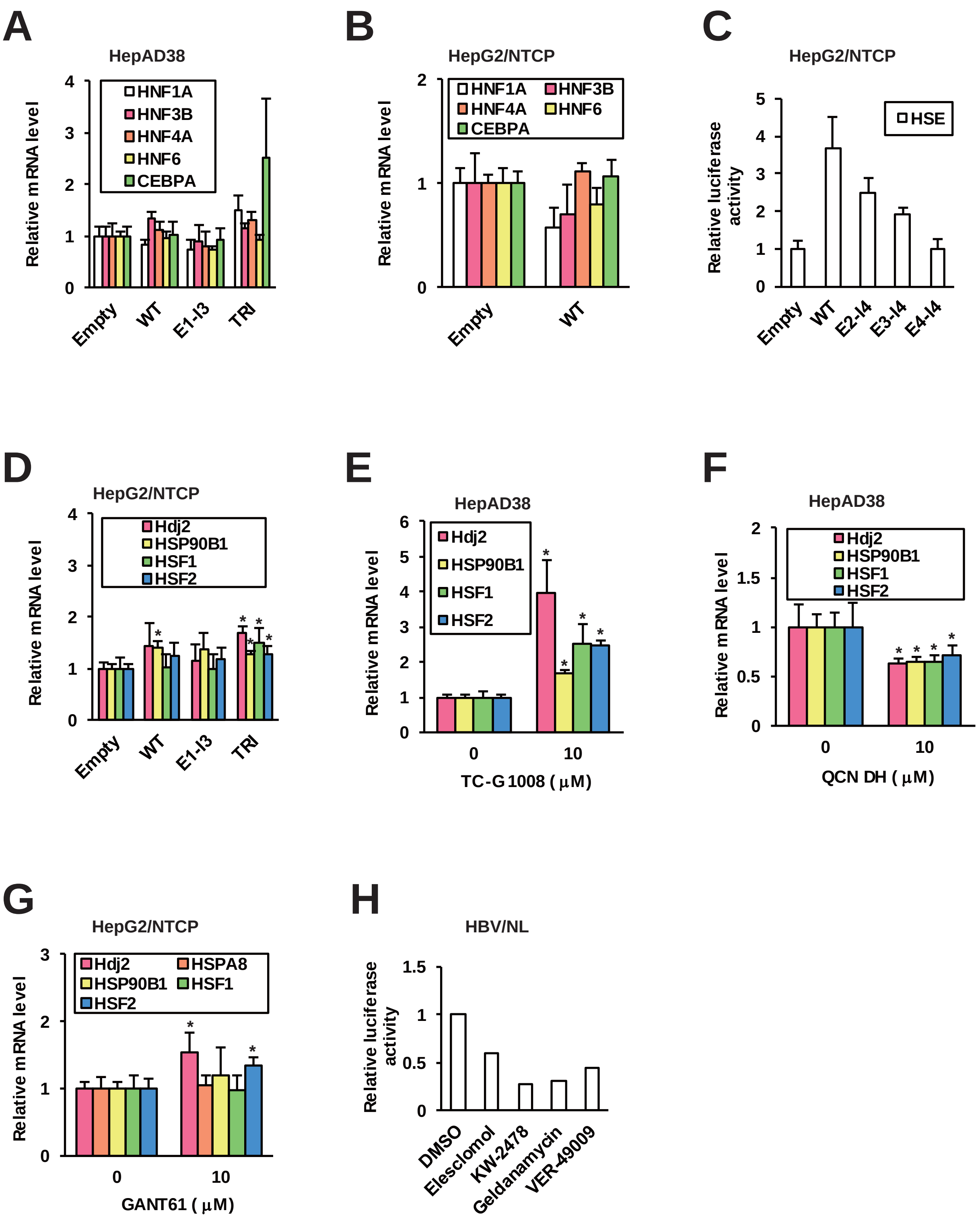

Supplementary Figure S3

Supplementary Figure Legends

Supplementary Figure S1. GPR39 expression. (A) Molecular models of GPR39 proteins. The structures of WT, truncated mutants E1-I3, E2-I4, E3-I4 and E4-I4 and the triple point mutant TRI with mutations, C108A, C191A and D313A in E2, E3 and E4, respectively. (B) The lentiviral expression of GPR39 proteins confirmed by western blot. C-terminally FLAG-tagged WT, E1-I3 and TRI with posttranslational modifications (PTMs) [1] were detected by anti-FLAG antibody (top panel). Unmodified GPR39 proteins according to molecular sizes were indicated by WT/TRI-3×FL and E1-I3-3×FL. The expression of ACTB was detected (bottom panel).

Supplementary Figure S2. Effects of GPR39 modulation on HBV and HSR. (A) Effects of TC-G 1008 on HBV in HepG2/NTCP cells. Twenty four hours after the infection with wild type HBV [2], HepG2/NTCP cells were treated with TC-G 1008 at 20 μM for 5 days. The relative levels of all and 3.5 kb HBV RNAs were measured by qRT-PCR. (B) The effects of TC-G 1008 in HepAD38 cells. After preculture without Tet for 7 days, the cells were treated with TC-G 1008 at 10 μM for 6 days in the presence of Tet. The relative mRNA level of GPR39 was measured by qRT-PCR. (C) Effects of GPR39 knockdown on viral promoter activities. HepG2/NTCP cells transfected with siRNAs to GPR39 for 3 days were transfected with reporter plasmids carrying viral promoters for 2 days, and the relative luciferase activities were measured.

Supplementary Figure S3. Effects of overexpression and pharmacological modulation of GPR39 on gene expression, HSR and HBV. (A) The effects of lentivirally overexpressed GPR39 proteins on liver-enriched transcription factors in HepAD38 cells. After preculture without Tet for 7 days, the cells were transduced with the lentiviruses for 6 days. The relative mRNA levels were measured by qRT-PCR. (B) The effects of lentivirally overexpressed GPR39 on liver-enriched transcription factors in HepG2/NTCP cells. After the transduction with the lentiviruses for the empty control and GPR39 for 2 days, the relative mRNA levels were measured by qRT-PCR. (C) Effects of truncated GPR39 proteins on HSR. The reporter plasmid pGL4.41 was cotransfected with the empty control p3xFLAG-CMV-14, WT or N-terminally truncated forms of GPR39, E2-I4, E3-I4 and E4-I4 into HepG2/NTCP-Myc cells for 48 h, followed by the measurement of relative luciferase activities. (D) The effects of lentivirally overexpressed GPR39 proteins on HSPs and HSFs in HepG2/NTCP cells. The cells were transduced with the lentiviruses for 2 days and the relative mRNA levels were measured by qRT-PCR. (E) The effects of TC-G 1008 on the expression of HSPs and HSFs in HepAD38 cells. After the treatment with TC-G 1008 at 10 μM for 6 days in the presence of Tet following preculture without Tet for 7 days, the relative mRNA levels were measured by qRT-PCR. (F) The effects of QCN on the expression of HSPs and HSFs. After preculture without Tet for 7 days, HepAD38 cells were treated with QCN DH at 10 μM for 6 days in the presence of Tet. The relative mRNA levels were measured by qRT-PCR. (G) Effects of GANT61 on the expression of HSPs and HSFs. After the treatment of HepG2/NTCP cells with GANT61 at 10 μM for 2 days, the relative mRNA levels were measured by qRT-PCR. (H) Antiviral activities of approved HSP inhibitors. The effects of elesclomol, KW-2478, geldanamycin and VER-49009 on HBV/NL in

our recent chemical screen [2] were demonstrated. Statistical significances with $P < 0.05$ to controls were indicated by asterisks.

Supplementary References

1. Holliday, N. D.; Holst, B.; Rodionova, E. A.; Schwartz, T. W.; Cox, H. M., Importance of constitutive activity and arrestin-independent mechanisms for intracellular trafficking of the ghrelin receptor. *Mol Endocrinol* **2007,** 21, (12), 3100-12.
2. Harada, K.; Nishitsuji, H.; Ujino, S.; Shimotohno, K., Identification of KX2-391 as an inhibitor of HBV transcription by a recombinant HBV-based screening assay. *Antiviral Res* **2017,** 144, 138-146.

Supplementary Table S1. The top five significant pathways connected to the GPR39-GSK3B axis and CRHR2

Pathway name	Entities				Reactions	
	Found	Ratio	P-value	FDR	Found	Ratio
Attenuation phase	12/47	0.003	3.33e-16	2.92e-13	3/5	3.94e-04
Regulation of HSF1-mediated heat shock response	15/113	0.008	6.66e-16	2.92e-13	9/14	0.001
HSF1-dependent transactivation	12/59	0.004	4.11e-15	1.20e-12	4/8	6.30e-04
Cellular response to heat stress	15/135	0.009	7.99e-15	1.75e-12	14/29	0.002
HSF1 activation	10/43	0.003	2.40e-13	4.20e-11	1/7	5.51e-04

Supplementary Table S2. The top 10 significant pathways connected to GALR2

Pathway name	Entities				Reactions	
	Found	Ratio	P-value	FDR	Found	Ratio
Post-chaperonin tubulin folding pathway	17/25	0.002	1.11e-16	2.22e-15	9/9	7.09e-04
RHO GTPase activate IQGAPs	22/36	0.002	1.11e-16	2.22e-15	5/5	3.94e-04
Formation of tubulin folding intermediates by CCT/TriC	17/30	0.002	1.11e-16	2.22e-15	2/2	1.57e-04
Cooperation of Prefoldin and TriC/CCT in actin and tubulin folding	18/37	0.003	1.11e-16	2.22e-15	6/6	4.72e-04
Prefoldin mediated transfer of substrate to CCT/TriC	13/29	0.002	1.11e-16	2.22e-15	2/2	1.57e-04
Carboxyterminal post-translational modifications of tubulin	18/52	0.004	1.11e-16	2.22e-15	6/6	4.72e-04
HSP90 chaperone cycle for steroid hormone receptors (SHR)	22/70	0.005	1.11e-16	2.22e-15	12/12	9.45e-04
Recruitment of NuMA to mitotic centrosomes	27/97	0.007	1.11e-16	2.22e-15	2/2	1.57e-04
Protein folding	24/106	0.007	1.11e-16	2.22e-15	22/28	0.002
Chaperonin-mediated protein folding	24/100	0.007	1.11e-16	2.22e-15	13/19	0.001

Supplementary Table S3. The top 10 significant pathways connected to the NPBWR1-MC4R axis

Pathway name	Entities				Reactions	
	Found	Ratio	P-value	FDR	Found	Ratio
Post-chaperonin tubulin folding pathway	17/25	0.002	1.11e-16	1.44e-15	9/9	7.09e-04
Formation of tubulin folding intermediates by CCT/TriC	17/30	0.002	1.11e-16	1.44e-15	2/2	1.57e-04
Prefoldin mediated transfer of substrate to CCT/TriC	12/29	0.002	1.11e-16	1.44e-15	2/2	1.57e-04
Carboxyterminal post-translational modifications of tubulin	18/52	0.004	1.11e-16	1.44e-15	6/6	4.72e-04
HSP90 chaperone cycle for steroid hormone receptors (SHR)	20/70	0.005	1.11e-16	1.44e-15	12/12	9.45e-04
Recruitment of NuMA to mitotic centrosomes	26/97	0.007	1.11e-16	1.44e-15	2/2	1.57e-04
MHC class II antigen presentation	19/148	0.01	1.11e-16	1.44e-15	21/26	0.002
Protein folding	18/106	0.007	1.11e-16	1.44e-15	20/28	0.002
Activation of AMPK downstream of NMDARs	19/34	0.002	1.11e-16	1.44e-15	2/3	2.36e-04
Cooperation of Prefoldin and TriC/CCT in actin and tubulin folding	17/37	0.003	1.11e-16	1.44e-15	4/6	4.72e-04

Supplementary Table S4. Primers for construction of plasmids

Name	Sequence
Nested-GPR39-F	AAGTCTTTGGACCTGGTAGC
Nested-GPR39-R	TGACGTTTTCTTAGGGCTGG
EcoRI-GPR39-F	TTCGAATTCGCCACCATGGCTTCACCCAGCCTCCCGGGC AGTGAC
XbaI-GPR39-R	AATTCTAGAAACTTCATGCTCCTGAAAACCATTCTCTGC
XbaI-GPR39-I3-R	AATTCTAGAGATGATGGTCTGCCTCCTGGCGGTCCTGCT
EcoRI-GPR39-E2-F	TTCGAATTCGCCACCATGGAGTTCTACAGCATCATCTGG AATCCCCTG
EcoRI-GPR39-E3-F	TTCGAATTCGCCACCATGGCCATGGGTACTGAGTACCCC CTGGTGAAC
EcoRI-GPR39-E4-F	TTCGAATTCGCCACCATGGCTGCGGCCAAACCCAAGCAC GACTGGACG
GPR39-C108A-F	CTACACCCTGTCCGCCAAGCTGCACACTTTC
GPR39-C108A-R	GAAAGTGTGCAGCTTGGCGGACAGGGTGTAG
GPR39-C191A-F	CCGGGGTCTCACTGCCAACCGCTCCAGCAC
GPR39-C191A-R	GTGCTGGAGCGGTTGGCAGTGAGACCCCGG
GPR39-D313A-F	CAAACCCAAGCACGCCTGGACGAGGTCCTAC
GPR39-D313A-R	GTAGGACCTCGTCCAGGCGTGCTTGGGTTTG
EcoRI-HBx-F1	TTCGAATTCGCCACCATGGCTGCTAGGGTGTGCTGCCAA CTGG
XbaI-HBx-R2	AATTCTAGAGGCAGAGGTGAAAAAGTTGCATGGTG
GPR39-INF-F	CCGGTCTCGAGAATTATGGCTTCACCCAGCCTC
GPR39-INF-R	TAACTCTAGAGGATCCTACTTGTCATCGTCATCC

Supplementary Table S5. Primers for qRT-PCR

Name	Sequence
HBV-ALL-F	GGGTGTGCTGCCAACTGGATCC
HBV-ALL-R	GTGAAGCGAAGTGCACACGGTC
HBV-3.5KB-F	GACCACCAAATGCCCCTATC
HBV-3.5KB-R	GATTGAGATCTTCTGCGACGC
GPR39-F	CGTCCAGCTACACCCTGTC
GPR39-R	CCAGACGAAGCCAATCAGC
HNF1A-F	AACACCTCAACAAGGGCACTC
HNF1A-R	CCCCACTTGAAACGGTTCCT
HNF3B-F	GGAGCAGCTACTATGCAGAGC
HNF3B-R	CGTGTTCATGCCGTTCATCC
HNF4A-F	CGAAGGTCAAGCTATGAGGACA
HNF4A-R	ATCTGCGATGCTGGCAATCT
HNF6-F	GAACATGGGAAGGATAGAGGCA
HNF6-R	GTAGAGTTCGACGCTGGACAT
CEBPA-F	GTCGGTGGACAAGAACAGCA
CEBPA-R	ATTGTCACTGGTCAGCTCCAG
CEBPB-F	GCACAGCGACGAGTACAAGA
CEBPB-R	TGCTTGAACAAGTTCCGCAG
Hdj1-F	CTCTGGACGGCAGGACGATA
Hdj1-R	TCTTGATGTCTGGGGAATCCTT
Hdj2-F	ACTGGAGCCAGGCGATATTAT
Hdj2-R	CTTCAACGAGCTGTATGTCCAT
HSPA8-F	ACCTACTCTTGTGTGGGTGTT
HSPA8-R	GACATAGCTTGGAGTGGTTCG
HSP90B1-F	GCTGACGATGAAGTTGATGTGG
HSP90B1-R	CATCCGTCCTTGATCCTTCTCTA
HSF1-F	CCATGAAGCATGAGAATGAGGC
HSF1-R	CTTGTTGACGACTTTCTGTTGC
HSF2-F	AGAATGAGTCCCTTTGGAAGGA
HSF2-R	TTCTTTTGGGCTCCATTAGTGTT
GADPH-F2	AAGGTGAAGGTCGGAGTCAAC
GADPH-R2	GGGGTCATTGATGGCAACAATA
